# Stable Expressed *DNMT3A* Mutants Predict a Poor Prognosis in Acute Myeloid Leukemia Patients Without Receiving Hematopoietic Stem Cell Transplantation

**DOI:** 10.1002/mco2.70151

**Published:** 2025-03-27

**Authors:** Xiang Zhang, Lixia Liu, Jiayue Qin, Xiong Ni, Jie Jin

**Affiliations:** ^1^ Department of Hematology The First Affiliated Hospital Zhejiang University School of Medicine Hangzhou Zhejiang People's Republic of China; ^2^ Department of Hematology Institute of Hematology Changhai Hospital Shanghai People's Republic of China

1

Dear Editor,

DNA methyltransferase 3A (*DNMT3A*) is wildly recognized as a tumor suppressor gene. Its deficiency leads to expanded hematopoietic stem cells (HSCs) pool, blocked HSCs differentiation, genomic instability, and a risk of malignant transformation in clonal hematopoiesis [1]. *DNMT3A* mutation (*DNMT3A* ^Mut^) is prevalent in adults, particularly in monocytic, and cytogenetically normal cases, affecting 25% of acute myeloid leukemia (AML) patients [1]. Although the distribution pattern of *DNMT3A* ^Mut^s has been well characterized in AML, its prognostic significance remains controversial.

To better understand the reasons behind *DNMT3A* ^Mut^ prognostic heterogeneity, we conducted a retrospective study, as detailed in two previous studies [2, 3]. Our findings show that *DNMT3A* ^Mut^s with stable expressed mutants are associated with poor prognosis in AML patients without receiving hematopoietic stem cell transplantation (HSCT). In this study, we enrolled 485 adult *de novo* AML patients, of whom 98 (20.2%) were found to have *DNMT3A* ^Mut^s. Our results indicate the distribution pattern of *DNMT3A* ^Mut^s and clinical characteristics of AML patients with these mutations were similar to previous reports. In our cohort, patients with *DNMT3A* ^Mut^s showed a relatively shorter overall survival (OS), relapse‐free survival (RFS) and disease‐free survival (DFS) (Figure 1A).

**FIGURE 1:**
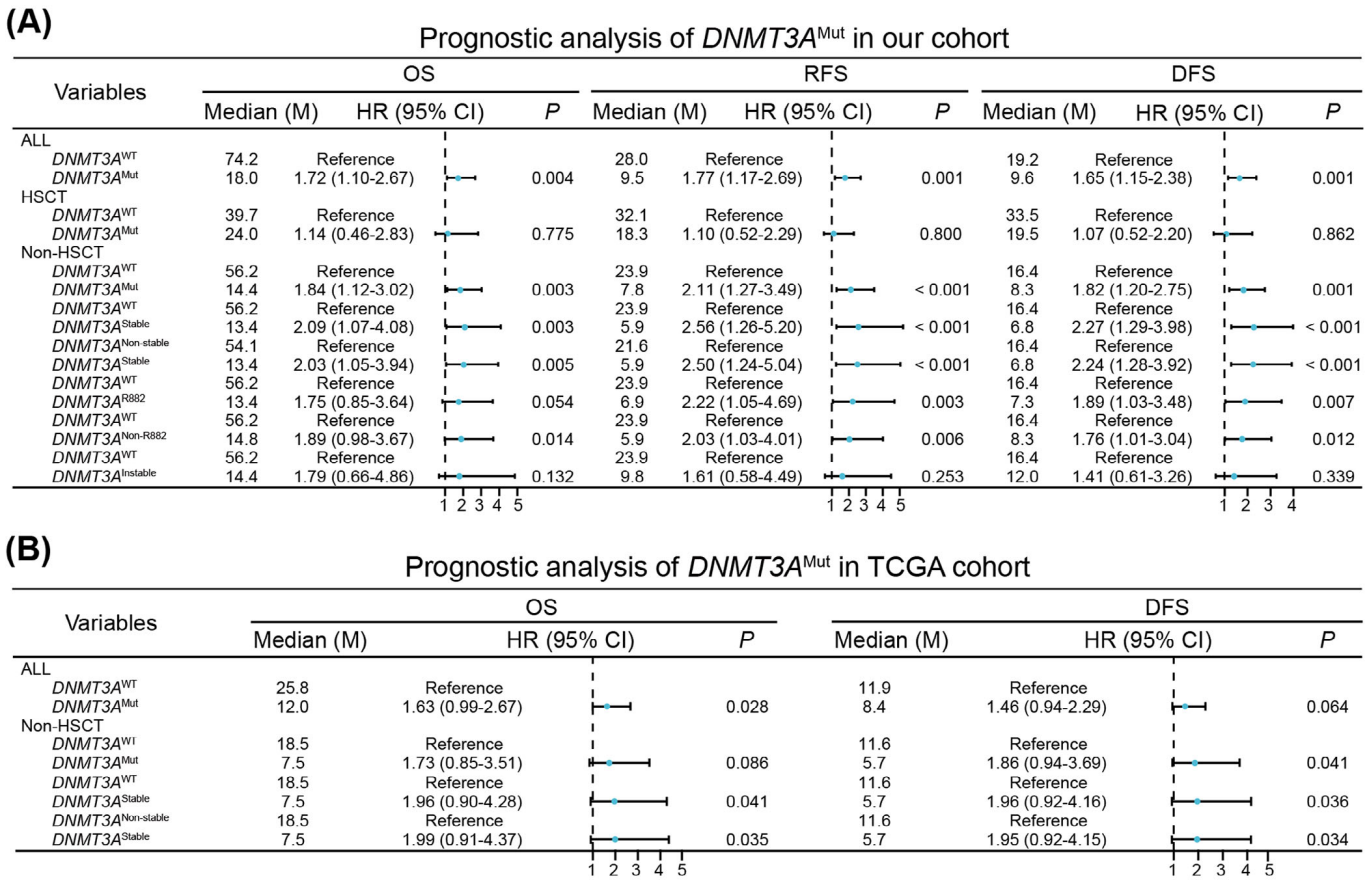
The Prognostic significance of *DNMT3A* mutation (*DNMT3A* ^Mut^) in acute myeloid leukemia (AML). (A) The influence of various *DNMT3A* ^Mut^ types on overall survival (OS), relapse‐free survival (RFS), and disease‐free survival (DFS) in our cohort. (B) External validation of the prognostic significance of *DNMT3A* ^Mut^ in The Cancer Genome Atlas (TCGA) cohort. HSCT, hematopoietic stem cell transplantation; HR, hazard ratio; CI, confidence interval; *DNMT3A* ^WT^, *DNMT3A* wild‐type; M, months.

We analyzed how different therapeutic strategies, with or without HSCT, affected the prognosis of various subgroups of *DNMT3A* ^Mut^ patients. In the HSCT group, *DNMT3A* ^Mut^ patients exhibited comparable OS, RFS, and DFS with *DNMT3A* wild‐type (*DNMT3A* ^WT^) patients (Figure 1A). Conversely, *DNMT3A* ^Mut^ patients showed poorer OS, RFS, and DFS in the non‐HSCT group, and the disparity between *DNMT3A* ^Mut^ and *DNMT3A* ^WT^ patients was more pronounced in the non‐HSCT group compared to the overall cohort (Figure 1A). Therefore, HSCT overcame the poor prognosis of *DNMT3A* ^Mut^, indicating *DNMT3A* ^Mut^ patients without receiving HSCT were primarily responsible for the unfavorable outcomes.

To further explore key factors contributing to poor prognosis of *DNMT3A* ^Mut^ patients, we focused on *DNMT3A* ^Mut^ types in the non‐HSCT group. Near 50% of *DNMT3A* ^Mut^ variants in AML are heterozygous *DNMT3A* ^R882^, the hotspot mutation [1]. As reported, *DNMT3A* ^R882^ played a dominant‐negative role against *DNMT3A* ^WT^ via formatting dimers, leading to genome‐wide hypomethylation [1]. However, *DNMT3A* ^non‐R882^ has been infrequently studied. Recently, Yung‐Hsin Huang et al. systematically studied protein stability of 253 disease‐associated *DNMT3A* ^Mut^ variants and their methyltransferase activity, and provided a comprehensive view on the implications of various *DNMT3A* ^Mut^ variants [4]. In our cohort, we identified 112 *DNMT3A* ^Mut^ variants. We first categorized *DNMT3A* ^Mut^ patients into two groups: *DNMT3A* ^R882^ and *DNMT3A* ^non‐R882^. Except for OS, both of *DNMT3A* ^R882^ and *DNMT3A* ^non‐R882^ patients a had worse RFS and DFS compared to *DNMT3A* ^WT^ patients, indicating that *DNMT3A* ^R882^ and *DNMT3A* ^non‐R882^ did not effectively distinguish between prognostic outcomes (Figure 1A). Accurately, nearly half of *DNMT3A* ^non‐R882^ variants were missense mutations, displaying similar mutant stability and diminished methyltransferase activity with *DNMT3A* ^R882^, so we further divided *DNMT3A* ^Mut^ patients into stable and instable *DNMT3A* ^Mut^ groups (Supplementary Materials and Methods). Notably, stable but not instable *DNMT3A* ^Mut^ patients exhibited worse OS, RFS and DFS compared to *DNMT3A* ^WT^ patients (Figure 1A). Additionally, we observed similar results when comparing stable *DNMT3A* ^Mut^ to nonstable *DNMT3A* ^Mut^ patients (nonstable *DNMT3A* ^Mut^ included both instable *DNMT3A* ^Mut^ and *DNMT3A* ^WT^) (Figure 1A). Thus, stable *DNMT3A* ^Mut^ was responsible for poor prognosis of *DNMT3A* ^Mut^ patients in the non‐HSCT group.

As stable *DNMT3A* ^Mut^ serves as a strong prognostic factor for AML patients, we were interested in exploring whether differences existed in distribution patterns and clinical characteristics between stable and instable *DNMT3A* ^Mut^. In our cohort, we identified stable *DNMT3A* ^Mut^ in 51 patients and instable *DNMT3A* ^Mut^ in 25 patients, while 22 *DNMT3A* ^Mut^ cases were undefined. Apart from a relatively higher frequency of *NPM1* mutation in stable *DNMT3A* ^Mut^ patients, the distribution pattern of stable *DNMT3A* ^Mut^ was similar to that of instable *DNMT3A* ^Mut^. Furthermore, compared to 412 nonstable *DNMT3A* ^Mut^ patients, 51 stable *DNMT3A* ^Mut^ patients presented a distribution pattern similar with *DNMT3A* ^Mut^ compared to *DNMT3A* ^WT^ patients. Definitely, stable *DNMT3A* ^Mut^ did not exhibit a specific distribution pattern. We also compared the baseline clinical characteristics of stable *DNMT3A* ^Mut^ with those of instable *DNMT3A* ^Mut^ or nonstable *DNMT3A* ^Mut^ patients. However, stable *DNMT3A* ^Mut^ patients did not obviously exhibit distinct clinical features. Although there were no significant statistical differences, the stable *DNMT3A* ^Mut^ group showed a relatively lower complete remission rate and a higher relapse rate compared to both instable *DNMT3A* ^Mut^ and nonstable *DNMT3A* ^Mut^ groups, regardless of whether all patients or non‐HSCT patients were considered, which possibly contributed to poor prognosis of stable *DNMT3A* ^Mut^ patients.

To determine whether stable *DNMT3A* ^Mut^ was an independent risk factor for poor prognosis in non‐HSCT patients, we divided patients into stable *DNMT3A* ^Mut^ group and nonstable *DNMT3A* ^Mut^ group, and displayed univariate analysis for OS, RFS, or DFS, respectively. Notably, stable *DNMT3A* ^Mut^ exhibited an adverse effect on OS, RFS and DFS. Following this, we conducted multivariate analyses, confirming that stable *DNMT3A* ^Mut^ was an independent adverse risk factor for OS, RFS or DFS in the non‐HSCT AML patients (Table S1).

Given its strong prognostic role, we sought to validate the clinical significances of stable *DNMT3A* ^Mut^ in external cohorts. Herein, we analyzed data from The Cancer Genome Atlas TCGA (TCGA) database, and found that stable *DNMT3A* ^Mut^ consistently indicates poor prognosis in the non‐HSCT patients (Figure 1B).

Although most evidences supported that *DNMT3A* ^Mut^ was a biomarker for poor prognosis in AML, the role of *DNMT3A* ^Mut^ in AML has not been widely accepted. In our cohort, *DNMT3A* ^Mut^ patients exhibited a shorter OS, RFS, and DFS in the entire cohort, consistent with results from most other reported cohorts. Most stable *DNMT3A* ^Mut^ variants are distributed across the methyltransferase domain, which is known to act as a dominant negative, resulting in decreased DNA methylation activity and a poor prognosis. HSCT eliminates leukemia cells, improves the hematopoietic function of the bone marrow, and enhances patient prognosis. A study has shown that HSCT improves outcomes of *DNMT3A* ^Mut^ AML patients [5], and our findings also indicate that HSCT reduces the prognostic gap between *DNMT3A* ^Mut^ and *DNMT3A* ^WT^ patients. In contrast, the prognostic disparity existed between *DNMT3A* ^Mut^ and *DNMT3A* ^WT^ patients in the non‐HSCT group. *DNMT3A* ^R882^, the hotpot mutation in *DNMT3A* ^Mut^ variants, was recognized as the primary factor contributing to the poor prognosis associated with *DNMT3A* ^Mut^ [1]. Nearly 50% of *DNMT3A* ^Mut^ variants are attributed to *DNMT3A* ^R882^, but significant heterogeneity also exits in *DNMT3A* ^non‐R882^ [1]. A recent study indicated that certain missense *DNMT3A* ^non‐R882^ variants also shared similar protein stability and methyltransferase activity features with *DNMT3A* ^R882^, thus, classifying *DNMT3A* ^Mut^ into *DNMT3A* ^R882^ and *DNMT3A* ^non‐R882^ was not accurate [4]. Herein, we classified *DNMT3A* ^Mut^ as stable or instable *DNMT3A* ^Mut^ according to mutant stability, and found that stable *DNMT3A* ^Mut^, rather than instable *DNMT3A* ^Mut^, was an independent predictor for relatively shorter OS, RFS and DFS. Furthermore, stable *DNMT3A* ^Mut^ primarily worsened the prognosis for AML genetic subtypes, indicating stable *DNMT3A* ^Mut^ was a dominant contributor for poor prognosis in the non‐HSCT patients. In the future, we aim to demonstrate the prognostic value of stable *DNMT3A* ^Mut^ through prospective multicenter clinical studies.

Collectively, stable *DNMT3A* ^Mut^, but not instable *DNMT3A* ^Mut^, should be regarded as a strong predictor of poor prognosis in AML patients without receiving HSCT, thereby providing insights into the mechanism underlying AML pathogenesis.

## Author Contributions

Xiang Zhang designed this study. Xiang Zhang and Xiong Ni collected clinical data and updated follow‐up. Xiong Ni and Jin Jie guided clinical managements for patients. Xiang Zhang, Lixia Liu, and Jiayue Qin displayed data analysis. Xiang Zhang and Jiayue Qin wrote the manuscript. Jie Jin provided critical comments on this study. Xiong Ni and Jie Jin revised the manuscript. All authors have read and approved the final manuscript.

## Ethics Statements

This study was approved by the ethical review committees of the First Affiliated Hospital of Zhejiang University School of Medicine (IIT20220659A) and Changhai Hospital (B2022‐035). All procedures in studies involving human participants were performed in accordance with the ethical standards of the institutional research committee and with the 1964 Helsinki Declaration and its later amendments. Written informed consent was obtained from these patients.

## Conflicts of Interest

The authors declare no conflicts of interest.

## Supporting information



Supporting Information

## Data Availability

The datasets used and/or analyzed during the current study are available from the corresponding author on reasonable request.
