# Supplementary material for: Stable Expressed DNMT3A Mutants Predict a Poor Prognosis in Acute Myeloid Leukemia Patients Without Receiving Hematopoietic Stem Cell Transplantation
Source: MedComm (2020). 2025 Mar 27;6(4):e70151. doi: 10.1002/mco2.70151 (PMC11949500; doi:10.1002/mco2.70151)
Supplement: Supplementary file 1 — Supporting Information [file MCO2-6-e70151-s001.docx]

**Supplementary Information**

**Stable expressed *DNMT3A* mutants predict a poor prognosis in acute myeloid leukemia patients without receiving hematopoietic stem cell transplantation**

Xiang Zhang^1#^, Lixia Liu^1#^, Jiayue Qin^1*^, Xiong Ni^2*^, Jie Jin^1*^

^1^Department of Hematology, The First Affiliated Hospital, Zhejiang University School of Medicine, Hangzhou, Zhejiang, People's Republic of China.

^2^Department of Hematology, Institute of Hematology, Changhai Hospital, Shanghai, People's Republic of China.

^#^These authors contributed equally to this work.

***Correspondence:** Jiayue Qin, jyqin@live.cn; Xiong Ni, xiongny1998@163.com; Jie Jin, jiej0503@zju.edu.cn.

**Materials and methods**

**Patients and targeted exome sequencing**

From 01/01/2010 to 31/08/2020, 485 adult *de novo* acute myeloid leukemia (AML) patients were involved in this study. 121 patients accepted hematopoietic stem cell transplantation (HSCT). All of patients were submitted to a targeted 185-gene exome sequencing (TES), based on next-generation sequencing at Acornmed Biotechnology Co., Ltd. The diagnosis, risky stratification, therapeutic strategies, response assessment, and method for TES analysis for patients were detailly described in our previous publications. The last follow-up was conducted at 09/07/2022.

**Definition for *DNMT3A* mutational types**

According to Yung-Hsin Huang et al.’ study, missense and in-frame insertion/deletion mutations generating stable mutants were defined as stable *DNMT3A* mutation (*DNMT3A*^Mut^), while missense and in-frame insertion/deletion mutations generating degradative mutants as well as nonsense and frameshift mutations were defined as instable *DNMT3A*^Mut^. Besides, those *DNMT3A*^Mut^ variants, which were not validated in this study, were recognized as undefined *DNMT3A*^Mut^, and they were excluded for analysis involving stability-based classification of *DNMT3A*^Mut^ variants.

**Statistical analysis**

Statistical analyses were carried out using R (version 3.5.1). Mann-Whitney U test was used for continuous variables. Chi-square test or Fisher’s exact test was used for categorical variables with adjustment for multiple testing using the Benjamini-Hochberg method. For analyzing the associations between different fusions and mutations, the false discovery rate correction was applied. Survival analysis was performed by the Kaplan-Meier method and differences assessed by the log-rank test. Variables with *P* < 0.1 by univariate analysis were entered into a multivariate analysis using a Cox proportional hazards model to identify the statistically integrate known clinical and genetic risk factors and potential confounders. Overall survival was calculated from the date of diagnosis to death or last follow-up. Relapse-free survival was calculated from the date of complete remission to relapse or death or last follow-up. Disease-free survival was calculated from the date of diagnosis to the relapse or death or last follow-up. A two-sided *P* < 0.05 was considered statistically significant.

**Table S1. Univariate and multivariate analysis for OS, RFS and DFS duration in non-HSCT AML patients.**

| **Variable** | **Univariate** | | **Multivariate** | |
| --- | --- | --- | --- | --- |
|  | **HR (95% CI)** | ***P*** | **HR (95% CI)** | ***P*** |
| **OS** |  |  |  |  |
| Stable *DNMT3A*^Mut^ (stable vs. non-stable) | **2.06 (1.24-3.43)** | **0.006** | **1.874 (1.023-3.435)** | **0.042** |
| Age (≥60 vs. <60) | **1.78 (1.22-2.60)** | **0.003** | - | - |
| *RUNX1::RUNX1T1* (positive vs. negative) | **0.21 (0.09-0.49)** | **<0.001** | **0.268 (0.107-0.672)** | **0.005** |
| *ASXL2* mutation (Mut vs. WT) | **0.38 (0.18-0.82)** | **0.014** | - | - |
| *FLT3*-ITD mutation (Mut vs. WT) | **1.82 (1.17-2.83)** | **0.007** | **1.797 (1.059-3.048)** | **0.030** |
| WBC (>9.5 vs. ≤9.5) | **1.58 (1.08-2.29)** | **0.017** | - | - |
| *WT1* mutation (Mut vs. WT) | 1.41 (0.84-2.36) | 0.191 | - | - |
| *BCOR* mutation (Mut vs. WT) | **1.86 (1.02-3.38)** | **0.043** | - | - |
| Chromosome 3 abnormality (positive vs. negative) | **3.20 (1.66-6.16)** | **<0.001** | **2.525 (1.193-5.342)** | **0.015** |
| Complex karyotype (positive vs. negative) | 1.70 (0.93-3.11) | 0.088 | - | - |
| *KDM6B* mutation (Mut vs. WT) | 0.58 (0.25-1.32) | 0.196 | - | - |
| *KIT* mutation (Mut vs. WT) | 0.66 (0.36-1.20) | 0.175 | - | - |
| *KMT2D* mutation (Mut vs. WT) | 1.41 (0.89-2.22) | 0.144 | - | - |
| *PTPN11* mutation (Mut vs. WT) | 1.61 (0.84-3.09) | 0.151 | - | - |
| *TP53* mutation (Mut vs. WT) | **3.31 (1.98-5.51)** | **<0.001** | **3.505 (1.944-6.321)** | **<0.001** |
| **RFS** |  |  |  |  |
| Stable *DNMT3A*^Mut^ (stable vs. non-stable) | **2.56 (1.55-4.22)** | **<0.001** | **2.204 (1.249-3.887)** | **0.006** |
| Age (≥60 vs. <60) | 1.43 (0.97-2.11) | 0.075 | - | - |
| *RUNX1::RUNX1T1* (positive vs. negative) | **0.49 (0.30-0.83)** | **0.008** | **0.514 (0.293-0.900)** | **0.020** |
| *ASXL2* mutation (Mut vs. WT) | **0.46 (0.24-0.88)** | **0.019** | - | - |
| *FLT3-ITD* mutation (Mut vs. WT) | **1.70 (1.06-2.73)** | **0.028** | - | - |
| WBC (>9.5 vs. ≤9.5) | **1.44 (1.00-2.08)** | **0.048** | - | - |
| *IDH1* mutation (Mut vs. WT) | 1.61 (0.92-2.82) | 0.095 | - | - |
| *IDH2* mutation (Mut vs. WT) | **0.41 (0.18-0.93)** | **0.033** | **0.304 (0.111-0.829)** | **0.020** |
| *TET2* mutation (Mut vs. WT) | **1.73 (1.14-2.62)** | **0.009** | - | - |
| *WT1* mutation (Mut vs. WT) | 1.43 (0.85-2.43) | 0.181 | - | - |
| Normal karyotype (positive vs. negative) | 1.37 (0.93-2.03) | 0.110 | - | - |
| **DFS** |  |  |  |  |
| Stable *DNMT3A*^Mut^ (stable vs. non-stable) | **2.28 (1.50-3.47)** | **<0.001** | **2.038 (1.219-3.406)** | **0.007** |
| Age (≥60 vs. <60) | **1.54 (1.13-2.12)** | **0.007** | - | - |
| *RUNX1::RUNX1T1* (positive vs. negative) | **0.40 (0.24-0.67)** | **<0.001** | **0.455 (0.262-0.788)** | **0.005** |
| *ASXL2* mutation (Mut vs. WT) | **0.39 (0.21-0.72)** | **0.003** | - | - |
| *FLT3-ITD* mutation (Mut vs. WT) | **1.55 (1.06-2.26)** | **0.023** | - | - |
| WBC (>9.5 vs. ≤9.5) | **1.44 (1.06-1.96)** | **0.019** | - | - |
| Chromosome 3 abnormality (positive vs. negative) | **2.49 (1.38-4.51)** | **0.002** | **2.301 (1.184-4.474)** | **0.014** |
| Complex karyotype (positive vs. negative) | 1.55 (0.95-2.54) | 0.081 | - | - |
| *PTPN11* mutation (Mut vs. WT) | 1.55 (0.89-2.68) | 0.120 | - | - |
| TP53 mutation (Mut vs. WT) | **2.11 (1.32-3.38)** | **0.002** | **2.075 (1.229-3.504)** | **0.006** |
| *IDH1* mutation (Mut vs. WT) | 1.45 (0.91-2.32) | 0.116 | - | - |
| *IDH2* mutation (Mut vs. WT) | 0.70 (0.41-1.18) | 0.182 | 0.526 (0.275-1.006) | 0.052 |
| *TET2* mutation (Mut vs. WT) | 1.40 (0.97-2.01) | 0.074 | - | - |
| *CBFβ::MYH11* (positive vs. negative) | 0.58 (0.28-1.17) | 0.128 | - | - |
| *CREBBP* mutation (Mut vs. WT) | 1.65 (0.97-2.80) | 0.066 | - | - |
| Hemoglobin (>82 vs. ≤82) | 1.26 (0.92-1.71) | 0.149 | - | - |

Abbreviations: OS, overall survival; RFS, relapse-free survival; DFS, disease-free survival; non-HSCT, non-hematopoietic stem cell transplantation; AML, acute myeloid leukemia; HR, hazard ratio; CI, confidence interval; *DNMT3A*^Mut^, *DNMT3A* mutation; Mut, mutation; WT, wild-type; WBC, white blood cell.
